# Fourier-Transform InfraRed Spectroscopy Can Quickly Type Gram-Negative Bacilli Responsible for Hospital Outbreaks

**DOI:** 10.3389/fmicb.2019.01440

**Published:** 2019-06-26

**Authors:** Daniel Martak, Benoît Valot, Marlène Sauget, Pascal Cholley, Michelle Thouverez, Xavier Bertrand, Didier Hocquet

**Affiliations:** ^1^Laboratoire d’Hygiène Hospitalière, Centre Hospitalier Régional Universitaire, Besançon, France; ^2^UMR 6249, Laboratoire Chrono-Environnement, Centre National de la Recherche Scientifique-Université de Franche-Comté, Besançon, France; ^3^Centre de Ressources Biologiques – Filière Microbiologique de Besançon, Centre Hospitalier Régional Universitaire, Besançon, France

**Keywords:** Fourier-transform infrared spectroscopy, FTIR, Gram-negative bacilli, hospital outbreak, bacterial typing

## Abstract

The typing of epidemic bacterial pathogens in hospitals relies on DNA-based, expensive, and time-consuming techniques, that are often limited to retrospective studies. However, the quick identification of epidemic pathogens in the routine of the microbiology laboratories would expedite infection control procedures that limit the contamination of new patients. IR Biotyper (Bruker Daltonics GmbH) is a new typing machine based on Fourier-transform infrared (FTIR) spectroscopy which generates spectra, aiming at typing the micro-organisms within 3 h. This technique discriminates the isolates by exploring the differences of the surface cell polysaccharides. In this work, we evaluated the ability of the FTIR spectroscopy to recognize Gram-negative bacilli clones responsible for hospital outbreaks. Isolates of *Pseudomonas aeruginosa* (*n* = 100), *Klebsiella pneumoniae* (*n* = 16), *Enterobacter cloacae* (*n* = 23), and *Acinetobacter baumannii* (*n* = 20) were typed by the reference methods Multi-Locus Sequence Typing (defining sequence types – STs) along with or without pulsed field gel electrophoresis (PFGE) (defining pulsotypes), and by FTIR spectroscopy. The congruence of FTIR spectroscopy clustering was compared to those of MLST and PFGE by Adjusted Rand index and Adjusted Wallace coefficient. We found that FTIR spectroscopy accurately clustered *P. aeruginosa*, *K. pneumoniae*, and *E. cloacae* isolates belonging to the same ST. The performance of the FTIR spectroscopy was slightly lower for *A. baumannii*. Furthermore, FTIR spectroscopy also correctly clustered *P. aeruginosa* isolates having a similar pulsotype. Overall, the IR Biotyper can quickly (in less than 3 h) detect the spread of clones of *P. aeruginosa*, *K. pneumoniae*, *E. cloacae*, and *A. baumannii*. The use of this technique by clinical microbiology laboratories may help to tackle the spread of epidemic clones by the quick implementation of infection control measures.

## Introduction

Gram-negative bacilli are involved in the four most frequent healthcare-associated infections (i.e., urinary tract infections, surgical site infections, pneumonias, and bloodstream infections) (Peleg and Hooper, 2010). Among these bacterial species, *Pseudomonas aeruginosa*, *Klebsiella pneumoniae*, *Enterobacter cloacae*, and *Acinetobacter baumannii* often acquire antibiotic resistance mechanisms allowing them to tackle the last generation treatments (Friedman et al., 2016). Infection control aims at limiting the spread of multidrug-resistant bacterial strains. Such outbreaks could be controlled with reinforced hygiene control measures that are quickly implemented after early detection of cross-transmissions (Woodford et al., 2011).

The quick detection of pathogen cross-transmissions in healthcare settings remains challenging despite the large choice of molecular typing methods available. Hence, multilocus sequence typing (MLST) defines sequence types (STs) by the combination of the alleles of fragments of seven housekeeping genes (Jolley and Maiden, 2014; Kimura, 2018). Matrix-assisted laser desorption ionization – time of flight mass spectrometry (MALDI-TOF MS) has successfully typed several bacterial subgroups (Siegrist et al., 2007; Berrazeg et al., 2013; Novais et al., 2014; Sauget et al., 2014; Cabrolier et al., 2015; Sousa et al., 2015) but its lack of robustness and the absence of a user-friendly software make it difficult to use routinely (Sauget et al., 2017). The highly discriminating whole genome sequencing (WGS) is becoming the new gold standard (Bertelli and Greub, 2013). However, Pulsed-field gel electrophoresis (PFGE), which clusters the isolates at the clonal level after macrorestriction of the total DNA (Talon et al., 1996), has been used for decades and is still used to investigate local outbreaks. These highly accurate techniques are laborious, time consuming and require trained staff. Hence, a quick and reliable typing technique that detects pathogen cross-transmissions in the routine of the clinical microbiology laboratory is still needed.

Fourier-Transform Infrared (FTIR) spectroscopy generates spectra based on the absorption of the infrared light by the different chemical components (i.e., lipids, proteins, or polysaccharides) of the whole bacterial cell. The absorption will be different depending on the components of the cell. The high variability of the surface cell makes the polysaccharide region (1300–800 cm^−1^) dominated by carbohydrates interesting for FTIR spectroscopy investigations. FTIR spectroscopy can identify species from the genus *Pseudomonas, Escherichia*, *Staphylococcus*, *Acinetobacter*, and *Klebsiella* (Quintelas et al., 2018). FTIR spectroscopy has also been used to type pathogens such as *Listeria monocytogenes* (Rebuffo-Scheer et al., 2007), *Staphylococcus aureus* (Johler et al., 2016), *Yersinia enterocolitica* (Kuhm et al., 2009), *Escherichia coli* (Dawson et al., 2014), or *K. pneumoniae* (Dinkelacker et al., 2018). However, this technique only started being implemented in microbiology laboratories recently (Novais et al., 2018).

In this work, we determined the discriminatory power of a marketed FTIR spectroscopy machine, using MLST and PFGE as typing references. We focused our study on Gram negative pathogens frequently involved in hospital outbreaks (i.e., *K. pneumoniae, P. aeruginosa*, *E. cloacae*, and *A. baumannii*). We also tested the benefit of the machine in the routine of the microbiology laboratories by simulating a prospective study.

## Materials and Methods

### Bacterial Isolates

We used 159 clinical isolates of *P. aeruginosa* (*n* = 100), *K. pneumoniae* (*n* = 16), *E. cloacae* (*n* = 23), and *A. baumannii* (*n* = 20) (Supplementary Table 1). The isolates were retrieved from outbreaks that occurred in hospitals from 11 French cities and are completed by outgroup isolates. All isolates were identified by MALDI-TOF MS (Microflex LT; Bruker Daltonik GmbH, Bremen, Germany) with a log value ≥ 2 according to the manufacturer’s recommendations and stored at −80°C at the Centre de Ressources Biologiques – Filière Microbiologique at the University Hospital of Besançon (Biobank BB-0033-00090) until further analysis.

### Molecular Genotyping

All isolates were typed by MLST using previously described schemes (Curran et al., 2004; Diancourt et al., 2005, 2010; Brisse et al., 2009; Miyoshi-Akiyama et al., 2013) (Supplementary Table 1). Of note, we used MLST ‘Pasteur’ scheme for *A. baumannii* typing. Although MALDI-TOF MS hardly differentiates *A. baumannii* from *A. pittii* or *A. nosocomialis*, the typeability of these isolates by *A. baumannii*-specific MLST scheme confirmed the identification of the isolates of our collection. In addition, 80 isolates of *P. aeruginosa* were genotyped by PFGE (CHEF DRIII, Bio-Rad) after total DNA restriction with *Dra*I (Roche Diagnostics) (Slekovec et al., 2012). We used Bionumerics software (Applied Math, Kortrijk, Belgium) to calculate a DNA similitude matrix based on Dice coefficient for pairwise isolate comparisons. The dendrogram was built with the UPGMA (unweighted pair group using arithmetic averages) hierarchical algorithm. *S. aureus* NCTC 8325 strain was the reference for inter-experiment comparison. The pulsotypes were determined according to international recommendations (Tenover et al., 1995).

### Sample Preparation for FTIR Spectroscopy Analysis

The samples were prepared in a room where temperature was 24 ± 1.5°C and hygrometry was 46 ± 8%. Bacterial isolates were cultured on Mueller-Hinton agar (Bio-Rad, Marne la Coquette, France) for 24 ± 1 h at 35 ± 2°C and a loopful of bacterial cells was suspended in 50 μL of ethanol. Suspensions were mixed before addition of 50 μL of water. The suspensions were homogenized and 15 μL of each suspension were dropped in triplicate on a silicon plate (Bruker Optics-Daltonics GmbH) and dried for 25 ± 5 min at 35 ± 2°C.

### Spectra Acquisition, Processing, and Analysis

Spectra were recorded in transmission mode in a spectral range of 4,000–400 cm^−1^ (mid-IR) using an IR spectrometer (Bruker Optics-Daltonics GmbH). Spectra were acquired, visualized, and processed by OPUS v7.5 software (Bruker Optics GmbH). Data from the area of polysaccharides (1,300–800 cm^−1^) were vector normalized, and the second derivative was used to amplify differences between isolates. IR Biotyper Client Software v1.5 (Bruker Daltonik GmbH) built dendrograms using Euclidian distance and average linkage clustering method.

### Clustering Concordance

The clusters obtained by MLST or PFGE were compared with those obtained by IR Biotyper with Adjusted Rand index (AR) and Adjusted Wallace coefficient (AW) with 95% confidence intervals (CI) (Carriço et al., 2006). AR compares partitions with no consideration for the reference method and evaluates the congruence between two typing methods. In comparison, AW takes into account inter-cluster distances and assesses the directional agreement between typing methods (Carriço et al., 2006; Pinto et al., 2008; Severiano et al., 2011). In other words, AR and AW equal to 1 reflect a perfect correlation between the two typing methods. The optimal cut-offs of each species was defined by maximizing AR between MLST and FITR results.

### Simulating a Prospective Study

To evaluate the FTIR spectroscopy in the routine of microbiology laboratories, we simulated a prospective study over 2 weeks. A total of 80 isolates of *P. aeruginosa* were analyzed. Sixty-one were retrieved from 13 independent outbreaks in four cities, and 19 non-epidemic isolates were used as outgroups. In order to simulate the daily work of a clinical laboratory, epidemic isolates (i.e., from the same ST and sharing the same pulsotype) were analyzed by FTIR spectroscopy in independent experiments ran on eight different days (Table 1).

**Table 1:** Temporal distribution of the isolates of *P. aeruginosa* typed by FTIR spectroscopy during 8 days.

*P. aeruginosa* isolates analyzed								
Pulsotype (ST)	Day 1	Day 2	Day 3	Day 4	Day 5	Day 6	Day 7	Day 8
A (ST308)		A1				A2	A3	A4,A5
B (ST175)	B1		B2		B3,B4			B5
C (ST395)	C1	C2,C3				C4		C5
D (ST244)	D1		D2, D3		D4			
E (ST708)					E1,E2	E3	E4	E5
F (ST560)	F1			F2	F3	F4,F5		
G (ST274)				G1, G2	G3		G4	G5
H (ST679)			H1, H2	H3	H4		H5	
I (ST111)		I1	I2				I3,I4	I5
J (ST207)		J1		J2		J3		
K (ST175)		K1		K2				K3
L (ST233)	L1	L2,L3				L4	L5,L6	
M (ST235)	M1, M2		M3	M4		M5		
Outgroups	O1,O2,O3	O4,O5	O6,O7,O8	O9,O10,O11	O12,O13	O14,O15	O16,O17	O18,O19

*The isolates of the different pulsotypes were typed on different days to simulate the reality of the clinical laboratory. For ease of reading, each pulsotype (A to M) was assigned with a different color (retrieved in Figure 3). Outgroups are numbered from O1 to O19.*

### Reproducibility

Since FTIR spectroscopy techniques are known to be very sensible to variations in culture media, incubation time, temperature, and hygrometry, we assessed the reproducibility of the technique by analyzing 20 isolates of *P. aeruginosa* four times independently on separate days (Dec. 8th, 2017; May 4th, 9th, and 15th, 2018).

## Results

### Microbial Typing

**FIGURE 1:**
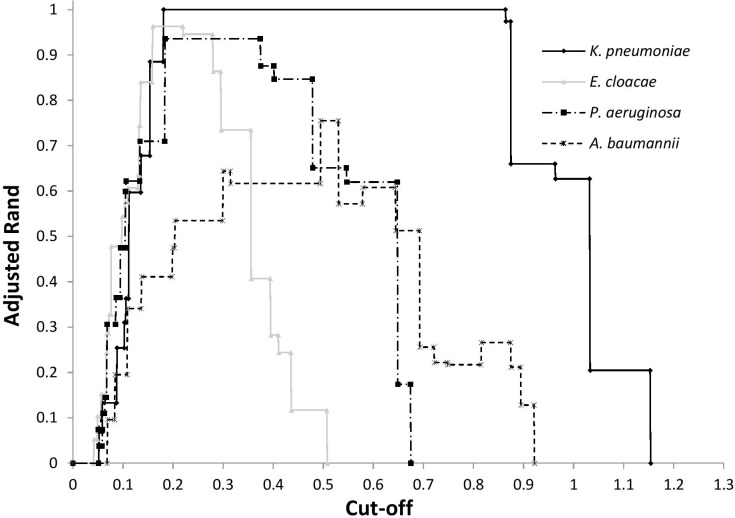
Adjusted Rand index according to cut-off for the clustering by FTIR spectroscopy of ST of *K. pneumoniae*, *P. aeruginosa*, *E. cloacae*, and *A. baumannii*.

**FIGURE 2:**
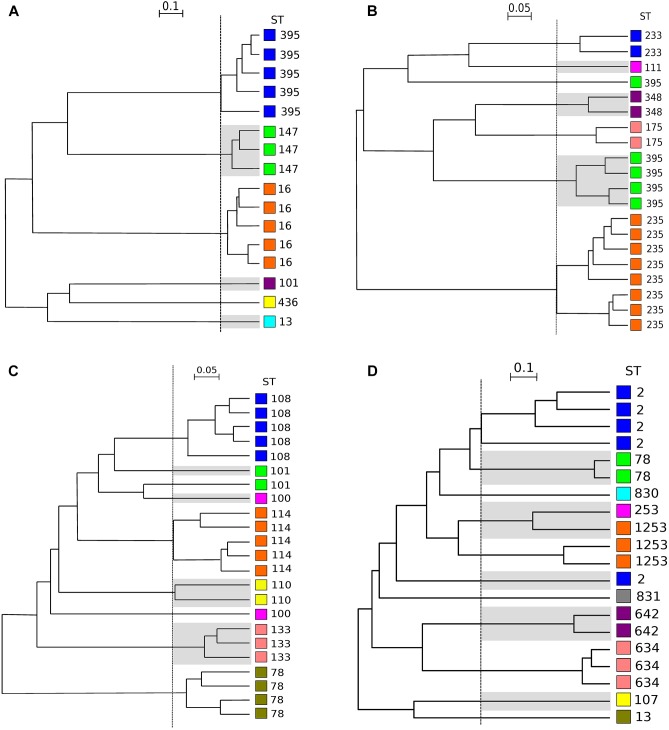
Dendrograms obtained by clustering FTIR spectra for *K. pneumoniae*, *P. aeruginosa*, *E. cloacae*, *A. baumannii*. The vertical dashed lines represent the cut-off values. **(A)** Clustering obtained for 16 isolates of *K. pneumoniae* (cut-off, 0.220). **(B)** Clusters obtained for 20 isolates of *P. aeruginosa* (cut-off, 0.221). **(C)** Clusters obtained for 23 isolates of *E. cloacae* (cut-off, 0.159). **(D)** Clusters obtained for 20 isolates of *A. baumannii* (cut-off, 0.495). ST is given for each isolate. For ease of reading, one out of two clusters are shaded and each ST was assigned with a different color (independent from those of Figure 3 and Table 1).

After FTIR spectra acquisition, dendrograms were built for each species. To assess the concordance between typings with MLST and FTIR spectroscopy, AR was calculated at different cut-off of clustering (Figure 1). For *K. pneumoniae*, the optimal cut-off ranged from 0.181 to 0.864 with perfect clustering (AR, 1.000) of the six different STs (Figure 2A). FTIR spectroscopy distributed the 16 isolates in six clusters corresponding to six different STs. For *P. aeruginosa*, the optimal cut-off varied from 0.184 to 0.374 (AR, 0.936) with one misclassified ST395 isolate (Figure 2B). The optimal cut-off for *E. cloacae* ranged from 0.159 to 0.219 (AR, 0.963) with two misclassified isolates (Figure 2C). For *A. baumannii*, the optimal cut-off ranged from 0.495 to 0.530 with a lower global concordance with MLST (AR, 0.755) for which three isolates were misclassified (Figure 2D). Hence, one ST2 isolate did not cluster with the other ST2 isolates and one ST1253 isolate clustered with a ST253 isolate.

Then, we evaluated the capacity of FTIR spectroscopy to detect a given ST type and vice versa by calculating AW (Table 2). For *K. pneumoniae*, *P. aeruginosa*, and *E. cloacae*, the AW of 1.000 indicated that two isolates clustered by FTIR spectroscopy always belonged to the same ST. The inverse was not true for *P. aeruginosa* and *E. cloacae* with the AW of 0.929 and 0.879, respectively. For *A. baumannii*, the concordance is lower than for other species. Hence, two isolates clustered by FTIR spectroscopy were most probably of the same ST (AW, 0.915) but the inverse is clearly not true with an AW of 0.642.

**Table 2:** Adjusted Rand (AR) and Adjusted Wallace (AW) coefficients with 95% CI calculated to compare FTIR spectroscopy (IRBT, Bruker Optics, Germany) and MLST for *K. pneumoniae*, *P. aeruginosa*, *E. cloacae*, and *A. baumannii*.

Species	Adjusted Rand	Typing method	Adjusted Wallace coefficients (95% CI) when compared with	
			FTIR	MLST
*P. aeruginosa*	0.936	FTIR		1.000 (1.000–1.000)
		MLST	0.879 (0.735–1.000)	
*K. pneumoniae*	1.000	FTIR		1.000 (1.000–1.000)
		MLST	1.000 (1.000–1.000)	
*E. cloacae*	0.963	FTIR		1.000 (1.000–1.000)
		MLST	0.929 (0.878–0.979)	
*A. baumannii*	0.755	FTIR		0.915 (0.830–1.000)
		MLST	0.642 (0.338–0.947)	

### Reproducibility

To assess the reproducibility of the FTIR spectroscopy, 20 isolates of *P. aeruginosa* were typed four times independently on separate days. The dendrograms built from these four independent experiments showed the same clustering (AR, 0.936) than that obtained for evaluation of microbial typing (Figure 2B), with only one ST395 isolate misclassified. These results indicate a good reproducibility of FTIR spectroscopy analysis.

### Robustness and Adaptation to the Clinical Lab Routine

**FIGURE 3:**
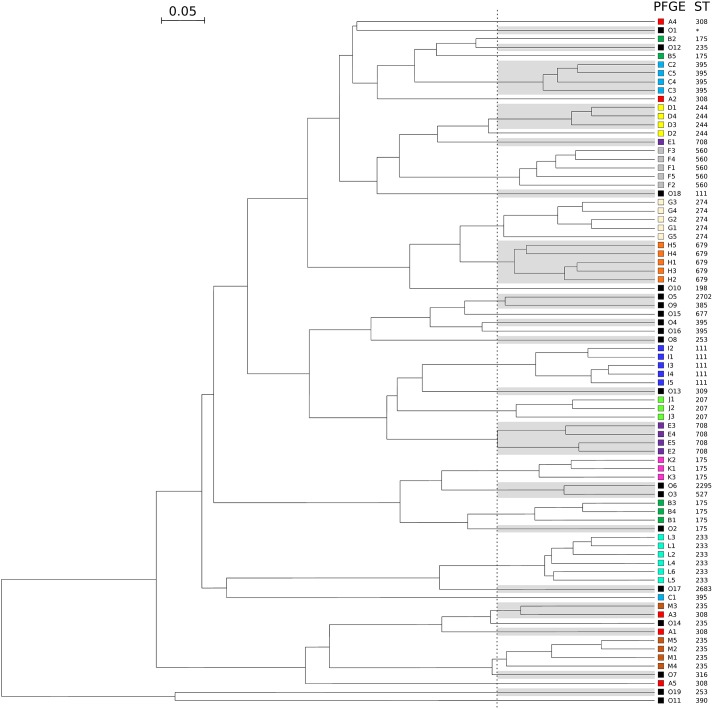
Dendrogram obtained by clustering the FTIR spectra of 80 isolates of *P. aeruginosa* during a simulated prospective study. The vertical dashed line represents the cut-off value. Pulsotypes and ST are given for each isolate. For ease of reading, one out of two clusters are shaded and each pulsotype was assigned with a different color (same than in Table 1). Outgroups are numbered from O1 to O19 and colored in black. The star indicates a ST not determinated.

To assess the ability of the FTIR spectroscopy to detect cross-transmissions of Gram-negative bacilli in the daily work of a clinical laboratory, 61 isolates of *P. aeruginosa* from 13 outbreaks were tested together with 19 outgroup isolates over 8 days (Table 1). Optimal cut-off was determined by calculating AR for all clusters. Figure 3 shows that FTIR spectroscopy adequately clustered the isolates of seven outbreaks (F-ST560, G-ST274, H-ST679, I-ST111, J-ST207, K-ST175, and L-ST233). For four other outbreaks (C-ST395, D-ST244, E-ST708, and M-ST235), one isolate was misclassified. For the outbreak B-ST175, two isolates were misclassified. The five isolates retrieved from the outbreak A-ST308 scattered throughout the FTIR dendrogram. Overall, the optimal cut-off for the clustering of epidemic *P. aeruginosa* isolates was 0.189 (AR, 0.824). This optimal cut-off was used to calculate AW, which indicated that two isolates clustered by FTIR spectroscopy were most probably from the same outbreak (AW, 0.965) while the opposite was less true (AW, 0.719).

## Discussion

### Discriminatory Power

We assessed here the discriminatory power of FTIR spectroscopy by typing four Gram-negative bacterial species implicated in hospital outbreaks. Overall, the discriminatory power of the FTIR spectroscopy is high for three of the four Gram-negative species tested (*E. cloacae*, *K. pneumoniae*, and *P. aeruginosa*), in agreement with other studies on other *Enterobacteriaceae* such as *Escherichia coli* (Davis et al., 2012; Sousa et al., 2013), *Klebsiella oxytoca* (Dieckmann et al., 2016), and *Yersinia enterocolitica* (Kuhm et al., 2009). We found that FTIR spectroscopy can type isolates of Gram-negative bacilli responsible for hospital outbreaks in 3 h. This contrasts with the current reference methods (i.e., MLST and PFGE) that require time and highly trained staff.

The results were compared to MLST and PFGE, two reference methods (Li et al., 2009; Larsen et al., 2012) for bacterial typing, by calculating AR and AW. FTIR spectroscopy correctly clustered isolates from the same ST for *K. pneumoniae* (AR, 1.000), *E. cloacae* (AR, 0.946) and *P. aeruginosa* (AR, 0.936). The performance of the FTIR spectroscopy was slightly lower for *A. baumannii* (AR, 0.755).

Using AW, we demonstrated that two isolates clustered by FTIR spectroscopy most likely shared the same ST. For *K. pneumoniae*, our results confirmed the high discriminatory power of FTIR spectroscopy reported in a recent study in which the authors found a good correlation between the clustering based on single nucleotide polymorphism and that based on FTIR spectroscopy (Dinkelacker et al., 2018).

Here, we report for the first time a high correlation for *E. cloacae* typing between FTIR spectroscopy and MLST, with only four isolates of two different STs which clustered separately, leading to a perfect AW but to a lower AR value (0.963) (Figure 2C).

Likewise, only one *P. aeruginosa* isolate was misclassified by FTIR spectroscopy. The genome of this isolate (namely DHS01) had undergone a 131-kb chromosomal deletion in comparison to those of the other isolates of the ST395 outbreak (Figure 2B; Petitjean et al., 2017). Such a large deletion, which encompasses genes involved in biofilm formation, virulence, adherence, and motility (Petitjean et al., 2017), could have modified the FTIR spectrum of the isolate thus accounting for the isolate misclassification.

The usefulness of FTIR spectroscopy is less obvious for *A. baumannii*. Despite the good correlation between MLST and FTIR spectroscopy reported in the litterature (Sousa et al., 2014), our results are a little bit less conclusive for this species than for the three other species tested. Since ST1253 is a single locus variant of ST2 (and consequently belongs to clonal complex CC2), we expected a clusterisation of isolates from these two STs, which were actually separate in the FTIR dendrogram. This presumably relies on the low discriminatory power of the Pasteur MLST scheme, which imperfectly reflects the high diversity of the CC2 (Graña-Miraglia and Castillo-Ramírez, 2019). To confirm the interest of FTIR spectroscopy in *A. baumannii* typing, FTIR spectroscopy has to be compared with more discriminant methods (i.e., WGS).

### Optimal Cut-Off Determination

For each dendrograms, IR Biotyper software automatically calculated a cut-off, which is the product of the Simpson’s index of diversity and the mean coherence of a parameter defined by the user (e.g., ST, pulsotype) (Hunter and Gaston, 1988). Since this cut-off was sub-optimal, we recalculated an optimal cut-off that provided the best AR. AR suitably determines the optimal cut-off since it compares the two typing methods without considering one as the reference (Hubert and Arabie, 1985). Unfortunately, we could not define a unique suitable cut-off for the four bacterial species tested. Hence, optimal cut-offs (i.e., which differentiated isolates at the ST level) depended on the bacterial species considered and ranged from 0.159 for *E. cloacae* to 0.495 for *A. baumannii* (Figure 1). For a given bacterial species, optimal cut-off probably depends on the genetic diversity of the population. The large range obtained for *K. pneumoniae* may be due to the low number of isolates tested (*n* = 16). Nevertheless, *K. pneumoniae*, *P. aeruginosa*, and *E. cloacae* had a similar optimal cut-off range (Figure 1). This cut-off range should be tested with other Gram-negative species to assess the possibility of setting up a cut-off suitable for different Gram-negative species.

### Reproducibility

Fourier-Transform Infrared spectroscopy is known to be a very sensible method with a high variability (Baker et al., 2014). We assessed the reproducibility of the method by repeating the analysis of 20 isolates of *P. aeruginosa* four times independently on separate days. FTIR spectroscopy clustering was highly reproducible with AR of 0.936 and AW always equal to 1.000. In particular, identical dendrograms were obtained by FTIR spectroscopy typing done 6 month apart, demonstrating the reliability of the method. The small variations of hygrometry (±8%) and temperature (±1.5°C) under which we tested the IR Biotyper are presumably crucial to keeping the performances of the method. In addition, the evaluation of the data exportability will require reproducibility tests on different machines.

### Prospective Situation

In order to test the robustness of the FTIR spectroscopy and its usefulness in the routine, we simulated a prospective study where *P. aeruginosa* isolates would be collected days after days. We found that FTIR spectroscopy accurately clustered isolates by outbreaks (as defined by PFGE; AR, 0.824), even though they were analyzed independently over 8 days. Hence, two isolates clustered by FTIR spectroscopy most probably belonged to the same clone (AW, 0.965), while the opposite was less true (AW, 0.719). In other words, FTIR spectroscopy clusters clonal isolates with a good specificity, but with a lower sensibility.

In contrast with the widely used typing methods (MLST, PFGE, and WGS) that only allow retrospective analysis, the FTIR spectroscopy could become a valuable tool that quickly identifies epidemic isolates in the flow of the laboratory routine. It will help seeking the sources of outbreaks and quickly implementing appropriate infection control measures. Although these measures are often implemented without molecular documentation, FTIR spectroscopy can help detect unsuspected outbreaks (of wild-type strains) or rule out wrong outbreaks (of isolates sharing an antibiotic resistance profile). FTIR spectroscopy could also help track environmental contamination sources by establishing a link between clinical and environmental isolates. Furthermore, FTIR spectroscopy can help see if a reinfected patient is colonized with the same or with a new strain. The marketed Bruker IR Biotyper has the potential to be implemented in a clinical lab routine with a quick and easy preparation of the samples and a user-friendly software.

### FTIR Spectroscopy

The performances of the technique could even be enhanced with the exploration of other spectral areas (proteins and lipids). Gold standard methods for bacterial typing such as MLST, PFGE, and WGS are DNA-based techniques while FTIR spectroscopy typing relies on carbohydrate composition. WGS data could help understanding the genomic variations between isolates that modify carbohydrate composition and subsequently impact the FTIR spectrum. In addition, other bacterial species should be tested to extend the list of species-specific cut-offs. Recommendations about culture conditions (i.e., media and growth conditions) and air parameters during sample preparation (i.e., hygrometry and temperature) would further help the implementation of the method in clinical laboratories.

## Conclusion

We found here that the FTIR spectroscopy accurately clustered *E. cloacae*, *K. pneumoniae*, *P. aeruginosa*, and *A. baumannii* isolates belonging to the same clones. The easiness of use, the short turnaround time, and the user-friendly software make FTIR spectroscopy adapted to the routine of the clinical laboratory. The quick bacterial typing with FTIR spectroscopy could help to tackle the spread of pathogens that threaten the weakest patients.

## Data Availability

The raw data supporting the conclusions of this manuscript will be made available by the authors, without undue reservation, to any qualified researcher.

## Author Contributions

DM, MS, XB, and DH conceived and designed the experiments. DM, PC, and MT carried out the experiments. DM and BV analyzed the data. DM, BV, XB, and DH wrote the manuscript.

## Conflict of Interest Statement

The authors declare that the research was conducted in the absence of any commercial or financial relationships that could be construed as a potential conflict of interest.
